# Combining Remote Temperature Sensing with in-Situ Sensing to Track Marine/Freshwater Mixing Dynamics

**DOI:** 10.3390/s16091402

**Published:** 2016-08-31

**Authors:** Margaret McCaul, Jack Barland, John Cleary, Conor Cahalane, Tim McCarthy, Dermot Diamond

**Affiliations:** 1 Insight Centre for Data Analytics, National Centre for Sensor Research, Dublin City University, Dublin 9, Ireland; margaret.mccaul@dcu.ie (M.M.); jackbarland@gmail.com (J.B.); 2 Carlow Institute of Technology, Carlow, Ireland; John.Cleary@itcarlow.ie; 3 National Centre for Geocomputation Ireland, Maynooth, Ireland; Conor.Cahalane@nuim.ie (C.C.); tim.mccarthy@nuim.ie (T.M.)

**Keywords:** in-situ sensing, sea-surface temperature, remote sensing, groundwater, salinity, nutrients, sensor networks

## Abstract

The ability to track the dynamics of processes in natural water bodies on a global scale, and at a resolution that enables highly localised behaviour to be visualized, is an ideal scenario for understanding how local events can influence the global environment. While advances in in-situ chem/bio-sensing continue to be reported, costs and reliability issues still inhibit the implementation of large-scale deployments. In contrast, physical parameters like surface temperature can be tracked on a global scale using satellite remote sensing, and locally at high resolution via flyovers and drones using multi-spectral imaging. In this study, we show how a much more complete picture of submarine and intertidal groundwater discharge patterns in Kinvara Bay, Galway can be achieved using a fusion of data collected from the Earth Observation satellite (Landsat 8), small aircraft and in-situ sensors. Over the course of the four-day field campaign, over 65,000 in-situ temperatures, salinity and nutrient measurements were collected in parallel with high-resolution thermal imaging from aircraft flyovers. The processed in-situ data show highly correlated patterns between temperature and salinity at the southern end of the bay where freshwater springs can be identified at low tide. Salinity values range from 1 to 2 ppt at the southern end of the bay to 30 ppt at the mouth of the bay, indicating the presence of a freshwater wedge. The data clearly show that temperature differences can be used to track the dynamics of freshwater and seawater mixing in the inner bay region. This outcome suggests that combining the tremendous spatial density and wide geographical reach of remote temperature sensing (using drones, flyovers and satellites) with ground-truthing via appropriately located in-situ sensors (temperature, salinity, chemical, and biological) can produce a much more complete and accurate picture of the water dynamics than each modality used in isolation.

## 1. Introduction

Understanding the inter-play of local events and global environmental processes is a frustratingly elusive but critically important goal, as it is fundamental to creating a deeper and more accurate understanding of these processes, and for ensuring that environmental legislation at global and local levels is made on an appropriately informed basis. For a restricted number of physical parameters (e.g., colour, temperature), global scale sensing can be achieved and dynamics visualised via satellite remote sensing. However, satellite sensing has also has significant limitations such as spatial resolution, low frequency pass-over cycle, inability to track sub-surface events, cloud cover preventing measurements, inability to directly sense detailed molecular information (except for the limited range of species that are directly accessible through spectroscopic methods), and need for specialist knowledge to convert data into accessible (visualized) information. That being said, satellite remote sensing is a very powerful means for accessing information at a global scale, and the capabilities of these platforms continues to improve. For example, the AVHRR Pathfinder Version 5.2 data set (PFV52) is a collection of global, twice-daily, 4 km grid resolution sea surface temperature data dating back to 1981 [1]. The European Space Agency Sentinel 2A satellite launched 23 June 2015, and Sentinel 2B (launch planned mid. 2016) will have a repeat cycle of 5 days (for the two satellites) and will provide access to a new range of powerful high-resolution remote sensing modalities [2]. 

The ideal scenario is to combine highly specific and localized chemical and biological information from in-situ sensor networks with the global reach of satellite remote sensing. However, the complexity and high cost of in-situ chemical and biological sensing platforms continues to restrict deployments to a relatively small number of widely separated autonomous devices, and manual grab sampling combined with centralized instrumentation is still extensively employed [3]. Appreciation that global scale environmental effects like climate change are driven by an aggregation of localized behaviours underscores the need for a combination of rich, localized and high-frequency data, with geographical scale information, if we are to understand the interplay between these local and global processes. In order to demonstrate the tremendous added value of blending remote spectral (temperature) data with in-situ sensed data augmented by more detailed chemical information obtained via grab sampling, we decided to examine submarine and intertidal groundwater discharge (SIGD) in the region of Kinvara Bay, Ireland. While this study is necessarily limited in geographical span and duration, the location is of significant interest, as a number of highly localised intertidal freshwater springs produce dynamic patterns of freshwater/sea water mixing in the inner bay. 

SIGD is of increasing global significance due to its role in transporting freshwater (and freshwater contaminants) to the ocean. The increasing anthropogenic impacts on the environment pose significant risks to the quality of aquatic systems, and karst systems (as exist in the Kinvara region) are particularly vulnerable to these effects. For example, intensive agricultural activities and sub-standard coastal sewage treatment works may cause increased contaminant and nutrient runoff that ultimately results in degradation of coastal waters. Previous groundwater monitoring programs have classified the Irish Western River Basin District (WRBD) and specifically the Kinvara-Gort groundwater body (GWB) to be high risk in this regard [4] and groundwater and nutrients entering the bay can remain for up to 10 days [5]. The Kinvara-Gort GWB is primarily underlain by limestone that includes large fissures, cracks, and tunnels that act as preferential flow paths for groundwater. To date, discharge from the western coast of Ireland has mainly been estimated using surface water runoff due to the difficulty in locating and quantifying SIGD [6]. The aim of this project was to investigate how complementary sensing modalities (Earth Observation satellite data, small aircraft multispectral imaging and in-situ sensing) could be used collaboratively to study the various SIGD occurrences in Kinvara Bay. 

We were particularly interested in tracking these mixing dynamics using temperature, as temperature can be accurately measured over large distances using thermal imaging from satellites and flyovers. The ability to track differing water types using temperature differences opens intriguing possibilities for environmental monitoring, as, by extension, it allows the distribution of water chemistry in the bay to be spatially mapped without the need for a dense deployment of (expensive) in-situ chemical sensors. To investigate this possibility, high-resolution thermal-imaging patterns obtained via synchronous flyovers were validated using in-situ temperature measurements. Simultaneous in-situ conductivity measurements provided a means to verify the relationship between water type (fresh water vs. sea water) and temperature, and grab samples would offer insights into the correlation of nutrient concentration with water type in the bay. 

## 2. Materials and Methods

### 2.1. Sample Site

Kinvara Bay (53.139° N 8.938° W) is located off the western coast of Ireland near the city of Galway. It is orientated in a NNW-SSE direction and opens to the southern edge of Galway Bay, which is connected to the Atlantic Ocean. Kinvara Bay is approximately 4.5 km long and covers a maximum area of 5.97 km^2^ during high tide. The average depth of the Bay is 4.2 m with an average daily tidal range of 3 m [7]. The area is part of the southeastern corner of the Western River Basin District (WRBD) of Ireland. Surrounding Kinvara Bay, the relief is mostly flat with no terrain more than 20 m above sea level. Dating back to 1861, the mean annual precipitation near Kinvara is about 1100 mm [8]. Under the assumption of negligible evapotranspiration during the winter months, mean annual evapotranspiration in the area has been estimated to be about 460 mm [9]. The mean annual potential recharge (precipitation minus actual evapotranspiration) is estimated to be in the range of 500–650 mm [10]. 

### 2.2. Hydrogeological Connection

**Figure 1:**
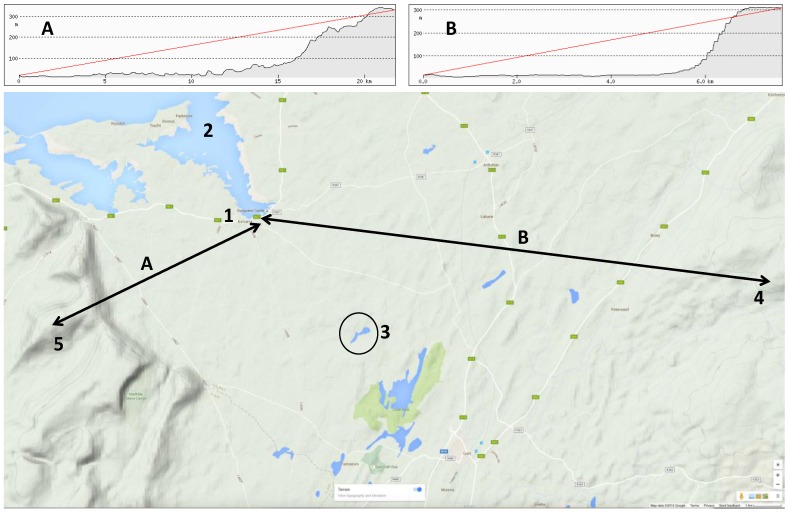
(**1**) Kinvara; (**2**) Kinvara Bay; (**3**) Caherglassuan Turlough; (**1**–**4**) Section A from Kinvara Eastwards towards Peterswell and the Slieve Aughty Region (**4**); (**1**–**5**) Section B from Kinvara Southwest towards Slieve Carran.

**Figure 2:**
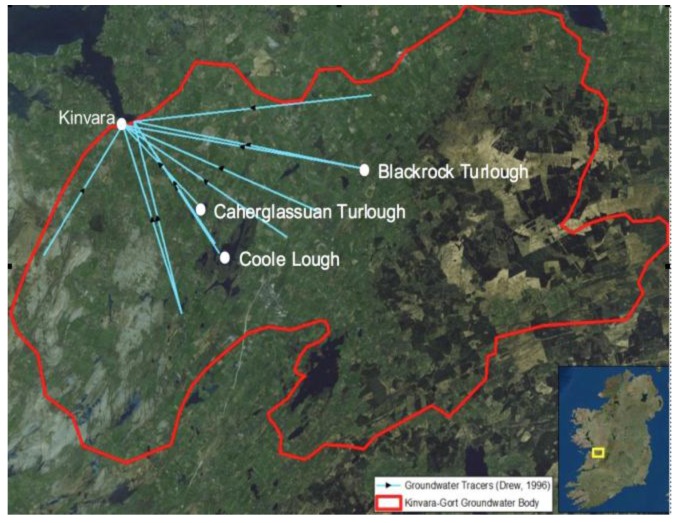
Hydrological connections to Kinvara Bay identified using groundwater tracers [11]. The blue lines represent subterranean pathways due to the Karst nature of the region that result in a number of inter-tidal discharges of cooler freshwater into the inner bay.

The dominant rock type underlying nearly half the land surface in Ireland is carboniferous limestone, which is susceptible to karstification. Lowland karst areas are characterized by rapid interaction between ground and surface waters as well as features that include losing and gaining streams, turloughs, springs, fissures, cracks, and tunnels. The Kinvara-Gort groundwater body (GWB) is one such area characterized by large fissures, cracks, and tunnels that act as preferential flow paths for groundwater. Dye tracer experiments have been used to track flow patterns and dynamics of the karst geology in the area, and these have identified three preferential flow paths at different depths, one of which is from Caherglassuan Turlough [11], see Figure 1 and Figure 2.

### 2.3. RGB Thermal Infrared Imaging

**Figure 3:**
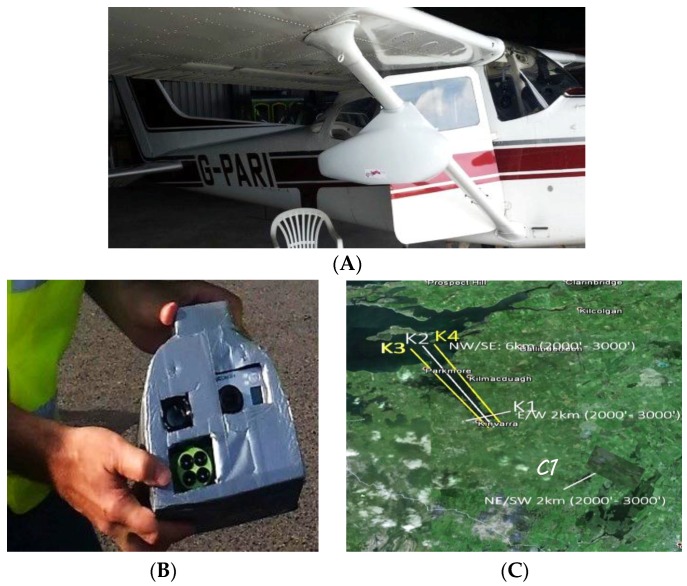
(**A**) Sensor pod mounted on the wing strut of a Cessna 172 light aircraft; (**B**) The NCG sensor pod used to acquire data; (**C**) Flight paths for aerial flyovers of Kinvara Bay and catchment area. K1 covers the inner bay while K2, K3 and K4 map the length of the bay from Kinvara to the mouth of the bay.

Thermal images of Kinvara Bay, Caherglassuan Turlough, and the surrounding area, were collected via flyovers by NUIM on 27 August 2015. Figure 3A–C shows the sensor pod mounted on the aircraft, details of the sensor pod itself, and the flight paths used. The data was georeferenced and mosaicked using ArcGIS [12]. The NCG sensor pod is comprised of four optical sensors:

1 A Nikon D800E SLR with a Zeiss T2 lens, capturing very high resolution RGB imagery enabling the creation of orthomosaics, point clouds and digital surface models thereby enabling visual comparisons with the thermal imagery.
2 A Tau 640 LWIR Thermal Imager, capturing data in the long-wavelength infrared portion of the spectrum from 7.5 μm to 13.5 μm.
3 An Airinov AgroSensor, primarily designed for vegetation surveys and recording imagery in the green, red, ‘red edge’ and NIR portions of the spectrum from approximately 0.53 μm to 0.83 μm. This can be used to create false colour orthomosaics, multispectral point clouds and digital surface models. A light metre is positioned on the top of the airplane cockpit to record changes in illumination during surveys for post processing.
4 A GoPro camera recording HD video.

The Tau 640 and the Nikon D800E recorded imagery during the aerial survey of Kinvara. Approximately 7 GB of data was captured during the 40-minute flights over the bay and turlough. Each overlapping RGB image collected during the survey was geotagged using two on-board GNSS receivers. An orthomosaic was then created from these overlapping images using the processing software Pix4D to eliminate mapping distortions. Subsequently, individual frames from the thermal camera were georectified by identifying a number of fixed points in the orthomosaic and applying a transformation polynomial.

### 2.4. In-Situ Data Acquisition

**Figure 4:**
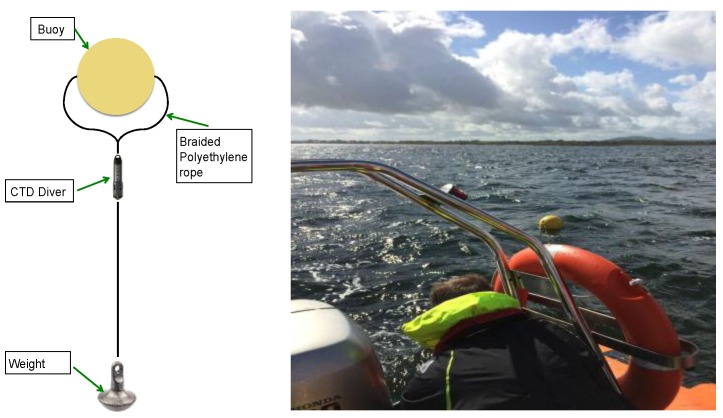
Schematic diagram of the sampling rig set up (**left**) showing the CTD Diver sensor attached to a float using a 2 mm braided polyethylene rope with a weight fixed at the bottom to keep the sensor in a vertical position. The float can be seen (**right**) slightly left of centre during a sensing transect of the bay.

**Figure 5:**
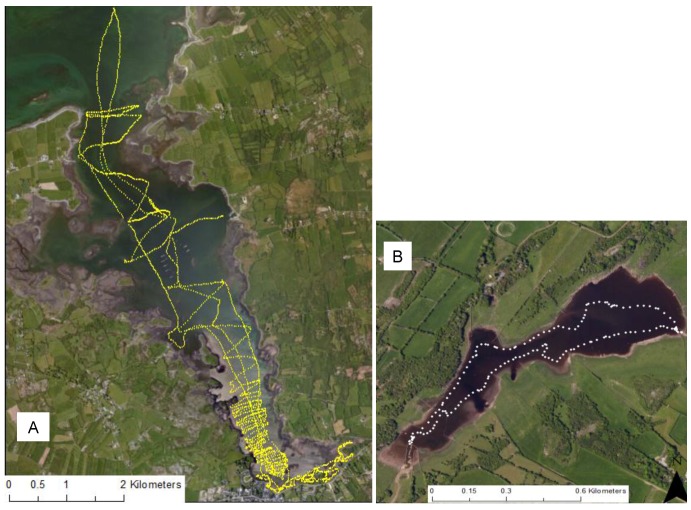
Location of in-situ data points collected over a four-day sampling campaign in (**A**) Kinvara Bay and (**B**) Cahergluassuan Turlough (25–28 August 2015).

The primary sampling campaign took place from 25 to 28 August 2015. The tidal range during this period was approximately 4.6 m measured from the tide gauge at Galway harbor. Data acquisition took place during high and low tide to enable tidal influences on mixing to be studied. Specific conductance and temperature measurements were made using a CTD diver (VWR technologies), with the diver mounted either on the side of the kayaks, or to a buoy, which was towed behind the rib. The CTD diver was attached to the float using a using a 2 mm thick braided polyethylene rope; with a weight to ensure the sensor was maintained in a vertical position (Figure 4). Boat speed, distance and position of this customized sampling rig from the boat were optimised prior to data acquisition to ensure that the sensor was held at a constant depth and to minimise mixing effects arising from the boat wake turbulence. The CTD diver logged data every second during deployment. A Garmin GPS device was included on-board to simultaneously log geographic coordinates, which were subsequently merged with the CTD data. Specific conductance measurements were converted to salinity prior to data analysis. Transects were performed to cover all areas accessible by boat as far north as the mouth of Kinvara Bay, with inaccessible shallower areas covered using the Kayaks (Figure 5).

### 2.5. Heat Map Visualization of in-Situ Data

To identify groundwater discharge sites and help inform future deployments and field campaigns, in-situ surface measurements of temperature and salinity were interpolated to create ‘heat map’ data visualisations of the entire bay. The inverse distance weighting (IDW) method available with the ArcGIS spatial analysis toolbox was used to estimate cell values based on nearby known values (i.e., when the area of interest is in close proximity to the collected data). Interpolation using IDW is most accurate when applied to a dense dataset that covers the minimum values and maximum expected values. This study meets both requirements with over 65,000 data points being collected over wide temperature and salinity ranges. 

### 2.6. Nutrient Analysis

Grab samples were collected at 40 point sources in Kinvara Bay at high and low tide and at Caherglassuan lough. The time and location of each sampling point was recorded using the Garmin unit. Samples were filtered through a 0.22 μm PVDF filter membrane and frozen on site for subsequent laboratory analysis using spectrophotometric techniques. Briefly, for Nitrite 1 mL of sample/standard solution was transferred to 1.5 mL Eppendorf vials, 50 μL of Griess reagent was added to the vials and the resulting solution incubated at ambient temperature (approx. 23 °C) for 20 min. For the direct determination of Nitrate, 100 μL of VCl_3_ was added to 900 μL of sample/calibration solution. Samples were incubated at 60 °C for 20 min. Phosphate analysis was preformed using equal volumes of yellow reagent and phosphate standard/sample. All solutions were measured on a PerkinElmer UV-VIS-NIR Spectrometer Lambda 900 (Perkin Elmer, Waltham, MA, USA) using a wavelength of 540 nm for nitrite and nitrate and 375 nm for phosphate. 

## 3. Results

### 3.1. Preliminary Screening

In this study, we were particularly interested in exploring whether there was a relationship between water temperature and salinity, and whether this would enable temperature to be used as a surrogate means for distinguishing water type (fresh water vs. sea water) in Kinvara Bay. The first step was to establish whether there was a difference between groundwater temperature and sea surface temperature (SST), and the extent of this difference over time. Groundwater temperature data from various boreholes sampled by the Environmental Protection Agency Ireland were compared to SST data acquired from the Marine Institute (MI) Ireland [13]. This showed that maximum temperature differences occurred in both the summer and winter months, when the inflow of cooler groundwater into warm near shore sea water results in plumes of low salinity water at the sea surface, around sites of groundwater discharge.

### 3.2. Landsat TIRS Data Acquisition

**Figure 6:**
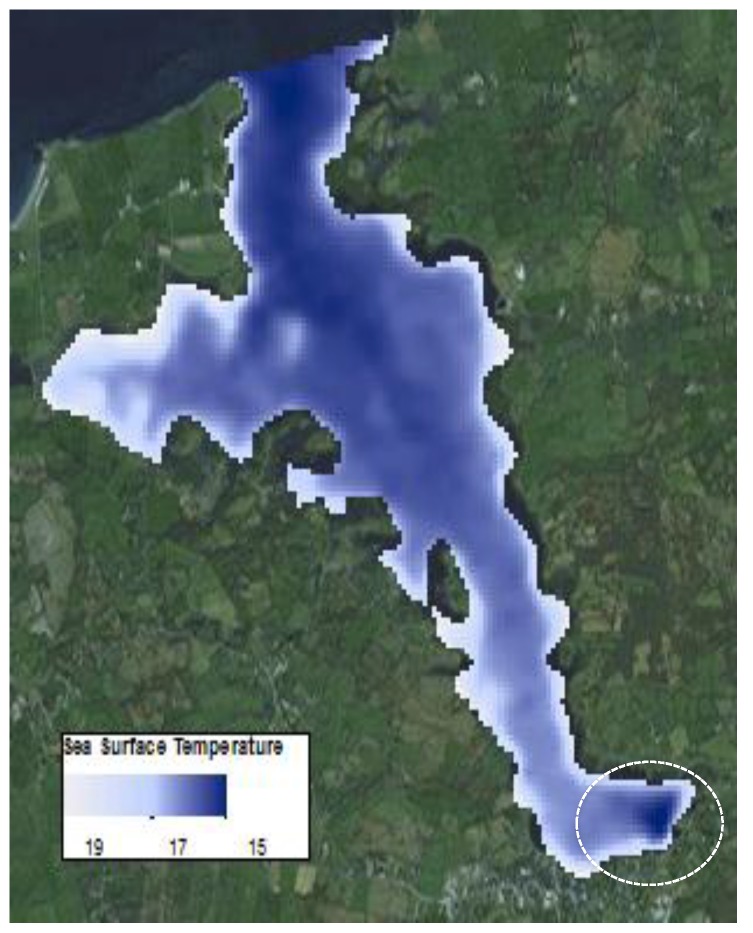
July 2013 sea surface temperature map of Kinvara Bay generated from Landsat 8 satellite imagery [14].

A total of 12 Landsat 8 thermal infrared sensor (TIRS) images of Kinvara Bay spanning the time period between January 2013 and May 2015 were acquired from data available from the US Geological Survey (USGS). The TIRS wavebands (bands 10 and 11) on Landsat 8 are sensitive in the electromagnetic regions between 10.3–11.3 micron and 11.5–12.5 micron [14]. The images used were mostly cloud free with a flyover time between 11:00 and 11:45 GMT (local time). As our focus was on the water temperature in the bay, land pixels were removed from the field of analysis, leaving only pixels that denoted water during high tide [15]. The processed images cover Kinvara Bay entirely and were subsequently used to create sea surface temperature and temperature anomaly maps as a function of time over a typical year. For example, Figure 6 shows averaged temperature patterns in the bay for the month of July 2013 we produced from data obtained from the US geological Survey. The colder freshwater emerging from sources in the inner bay are clearly visible at the bottom right of the image. This pattern is consistent with the in-situ temperature measurements and drone thermal imaging patterns.

### 3.3. Sea Surface Temperature and Salinity Maps from in-Situ Sensing

**Figure 7:**
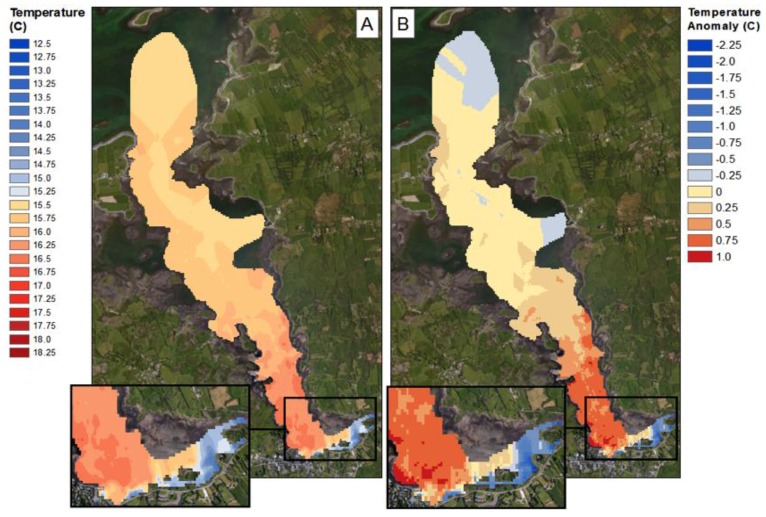
Sea surface temperature map of Kinvara Bay interpolated from in-situ data measurements (**A**); Temperature anomaly map showing surface temperature divergence from the mean (**B**).

**Figure 8:**
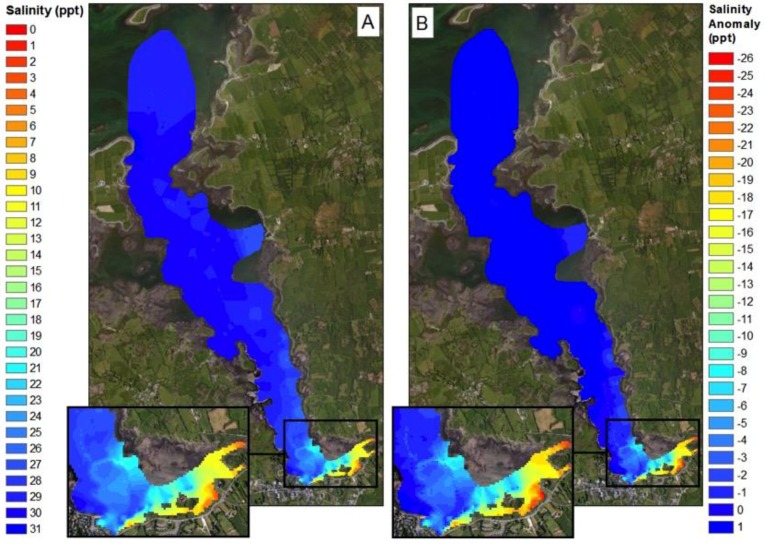
Sea surface salinity map of Kinvara Bay interpolated from in-situ data measurements (**A**); Salinity anomaly map showing surface salinity divergence from the mean (**B**).

The SST values obtained from the in-situ sensing data clearly show cold plumes of water in near shore waters along the southern coastline of Kinvara Bay (Figure 7 and Figure 8). Data collected during the field campaign show a range in temperature from a minimum of 13.5 °C (Southeastern part of the bay) to a maximum of 17.1 °C, with an average of 15.9 °C. On average, groundwater temperature in Ireland varies from 9.5 to 12 °C [8], and this is further supported by borehole records [5]. The minimum surface temperature observed within the SST maps occurs coincides with the plumes of cold groundwater. To better illustrate the significance of these cold-water plumes, temperature anomaly (TA) maps were produced that detail the decrease (or increase) in surface temperature in relation to the average surface temperature of Kinvara Bay. The TA values range from −2.25 to +1.0 °C as illustrated in Figure 7. 

**Figure 9:**
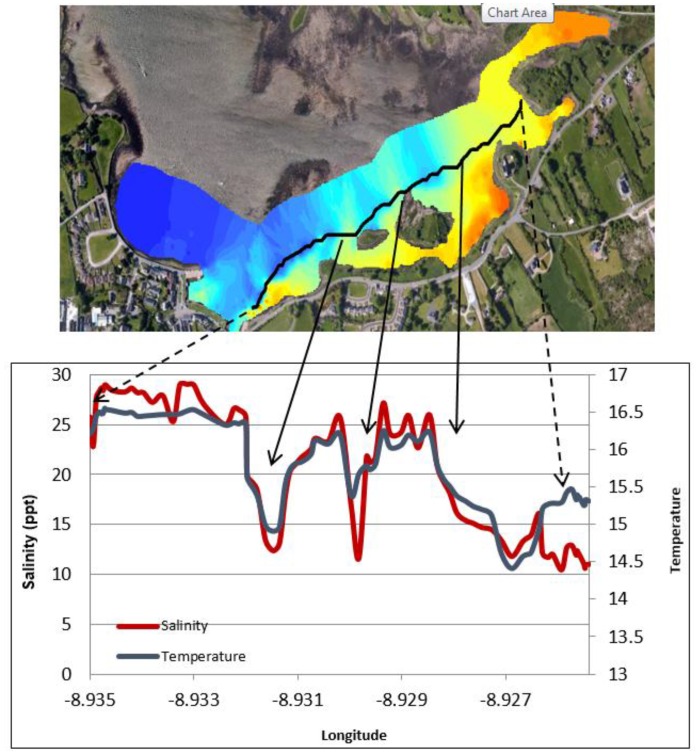
Temperature and salinity transect, stretching left to right from Kinvara pier (west) to Dunguaire Castle (east). The effect of individual cold-water plumes is clearly evident in the highly correlated features present in the salinity and temperature data.

The TA maps help to put the temperature anomalies in context and also allow comparison of the relative differences from one plume to another. Using TA maps and an understanding of the surrounding geology, interpretations of the location and extent of groundwater discharge can be made. The largest negative TA values were located directly west of Dunguaire Castle and extend east along the shoreline towards the town of Kinvara. This is consistent with visual field evidence of groundwater discharge at these locations and is highly correlated with salinity anomaly patterns (Figure 8). This correlation is strikingly illustrated in Figure 9, which shows coincident features in temperature and salinity data associated with the locations of several groundwater springs.

### 3.4. Tidal Influences

Over the four-day sampling campaign, there was an estimated tidal range of 4.6 m. As expected, this had an impact on the mixing dynamics of the freshwater plumes. The trends at high and low tide are similar but extend further north towards the mouth of the bay during low tide (Figure 9). At low tide, water depth is at a minimum, allowing freshwater inputs to have a greater influence on mixing in the southern portion of the bay. This is supported by the 0.5 °C decrease in water temperature during low tide and a decrease in salinity of 9 ppt. At both high and low tide, the strongest freshwater signature occurs near the castle, suggesting this is the largest source of freshwater. 

**Figure 10:**
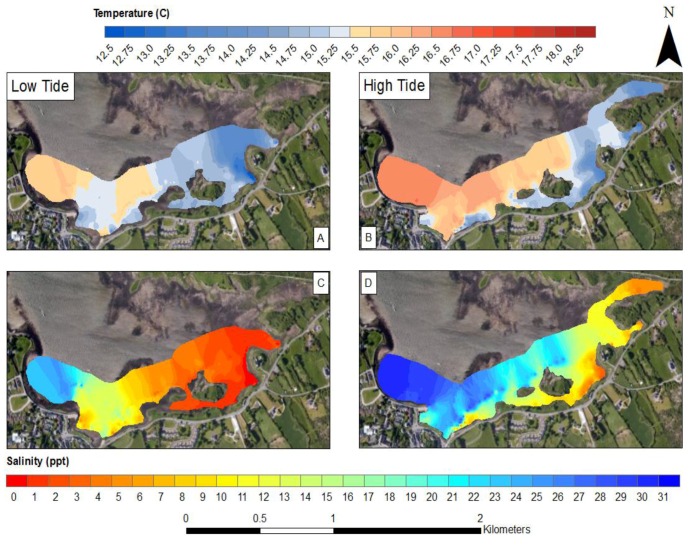
Sea surface temperature maps of southern Kinvara Bay interpolated from in-situ data measurements at low tide (**A**) and high tide (**B**); Sea surface salinity maps at low tide (**C**) and high tide (**D**).

**Figure 11:**
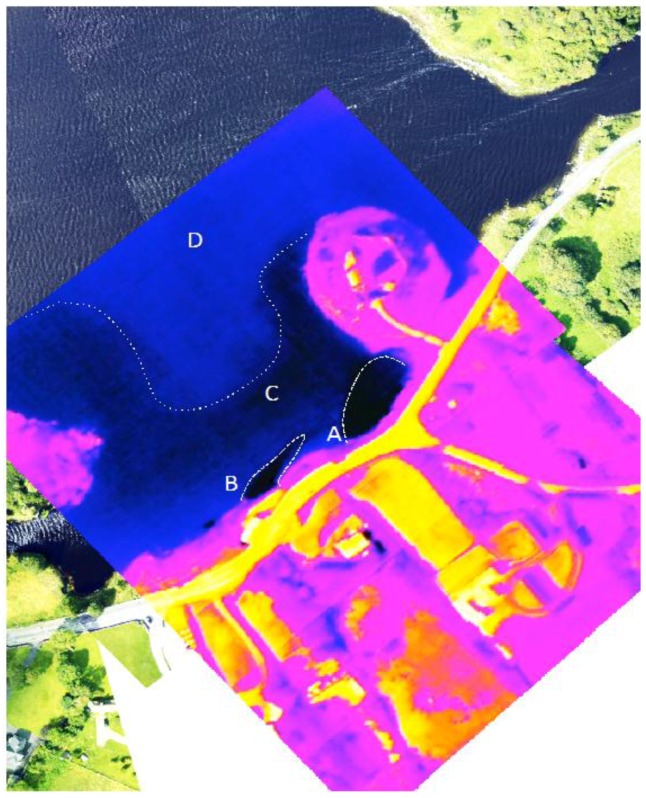
Thermal Imagery of Kinvara Bay collected using LWIR Thermal Imager interpolated with high-resolution imagery collected using a Nikon D800E SLR camera. Colder freshwater sources are clearly visible near the shore (**A**,**B**) as is a more extensive area of cooler water (**C**) which gradually merges with warmer water (**D**).

**Figure 12:**
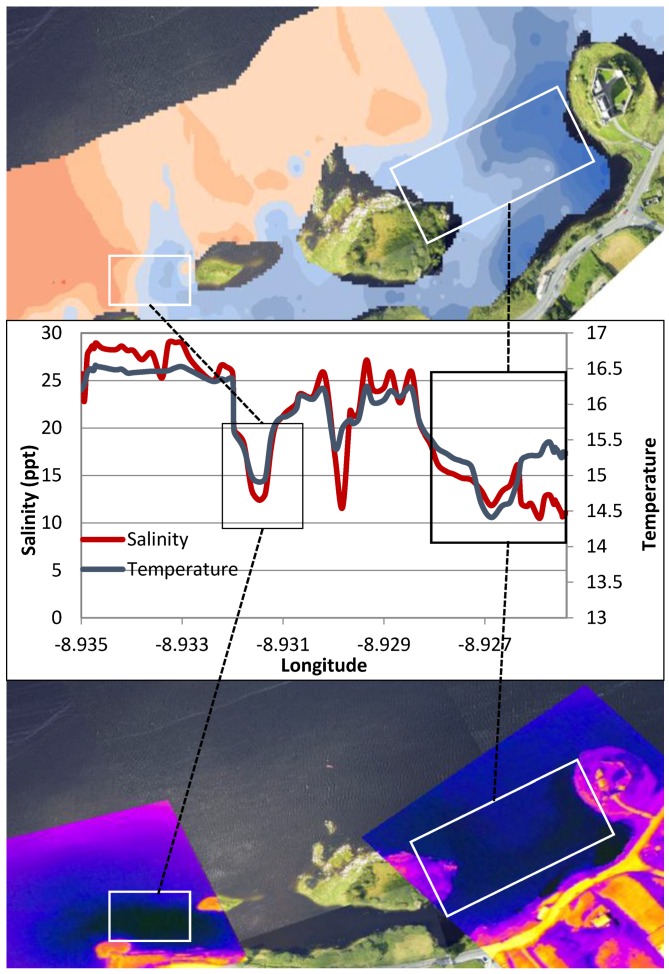
Sea surface temperature map from in-situ measurements (**top**) showing regions of cooler water (dark blue) in the inner bay. Temperature and salinity profiles (**middle**) plotted along a transect that extends from the town of Kinvara (left) to Dunguaire Castle (right). Temperature and salinity profiles are highly correlated and clearly identify lower temperature features consistent with the location of known fresh water sources. These features are also clearly visible in the thermal IR images obtained from the flyover (**bottom**).

The interpolated data indicate similar gradients in temperature and salinity exist across the bay, particularly in the southern half (Figure 8, Figure 9, Figure 10, Figure 11 and Figure 12). A longitudinal profile of sea surface temperature and salinity (Figure 9) reveals three distinct features in both temperature and salinity that correspond to the known locations of freshwater springs. These features also appear as cold-water plumes in the thermal images obtained from the fly-over data for which the main temperature features have been identified and labeled in Figure 11. A comparison of these data sources is presented in Figure 12. 

### 3.5. Caherglassuan Turlough

**Figure 13:**
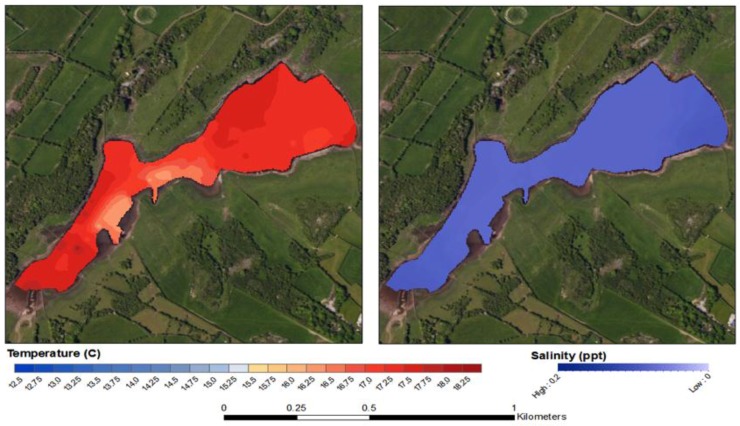
Surface temperature map (**A**) and Salinity map (**B**) of Caherglassuan Turlough.

**Figure 14:**
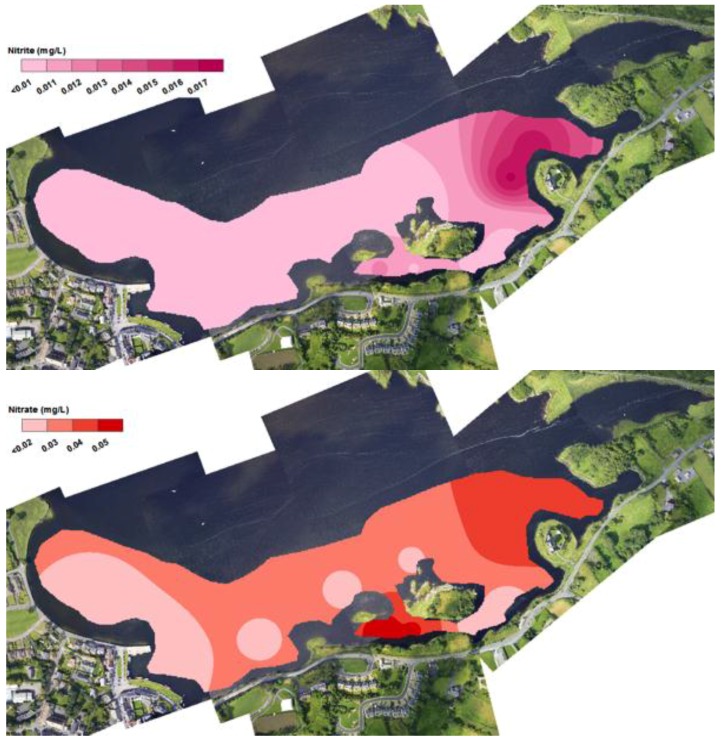

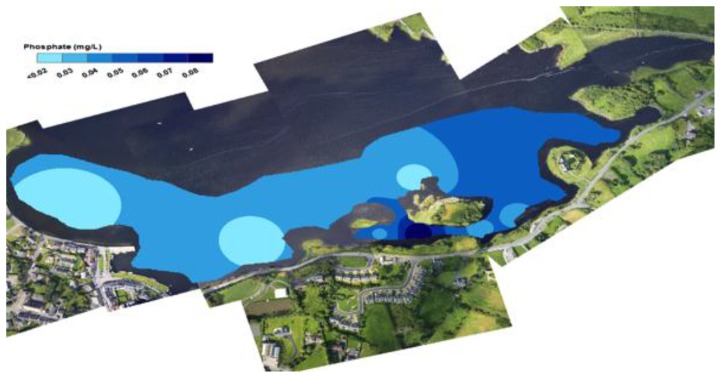
Interpolations of Nitrite (**top**), Nitrate (**middle**) and Phosphate (**bottom**) within Kinvara Bay at low tide during the sampling campaign (25–28 August 2015).

According to previously published research [4,8,11], the Caherglassuan Turlough is hydrologically connected to Kinvara Bay via a combination of conduits, fissures and cracks that are capable of transporting large volumes of groundwater. However, despite being inter-connected, results obtained during the sampling period show that two bodies of water display very different physical and chemical characteristics, see Figure 13 and Figure 14. On average, the temperature of the turlough is 2–3 °C warmer than the bay water and is well mixed with little spatial variation (Figure 13). Salinity values are near 0 ppt throughout the turlough, which supports the contention that there was no transfer of seawater from the bay to the lough through the connecting channels during the sampling period. Interestingly, although the turlough water temperature is higher than the seawater, by the time it emerges in the bay, the freshwater is cooler than the seawater.

In addition to groundwater discharge, freshwater transfer to the bay can occur by other mechanisms, such as surface runoff and river discharge. Therefore, it cannot be assumed that the thermal and salinity signatures observed from initial analysis are solely due to groundwater discharge. However, the consistent patterns and locations of significant features across the in-situ salinity and temperature measurements, and the temperature imaging data suggest that (for the inner bay at least) these springs are the main sources of freshwater. It is also worth noting that the ability to visualise freshwater movements in the bay depends significantly on the lower density of freshwater compared to seawater, which results in the freshwater locating preferentially in the surface layer [16]. The groundwater-seawater interface that occurs in the southern (inner) part of Kinvara Bay is therefore an ideal scenario to test these strategies for tracking water movement patterns. In contrast, freshwater-seawater interfaces arising from surface sources such as rivers in all probability would not present differences in temperature of the same extent, as the relatively low temperatures of the fresh water observed in this study appear to be associated with subterranean cooling. 

### 3.6. Nutrient Analysis

SGIDs have long been identified as possible conduits for nutrient loading in coastal oceans [6,9,16,17,18,19]. Kinvara Bay is particularly vulnerable to this effect due to the karstified landscape and the hydrogeological connection from nutrient bearing freshwater sources to the bay. Nutrient concentrations within Kinvara Bay and Caherglassuan lough reported in this study are an average of 10 measurements taken at each sampling location during the sampling campaign (25–28 August 2015), see Figure 5. Interestingly, nutrient concentrations within the inner bay were found to be lower at low tide than at high tide (see Figure 13 and Figure 14), possibly because the incoming tide inhibits movement and mixing (dilution) of the freshwater into the lower bay. Furthermore, in general, nutrient concentrations were consistently higher in samples taken at locations identified SGD sources, at both high and low tide, compared to non-freshwater sampling points [16,20].

Nutrient levels in Caherglassuan lough were relatively uniform in samples taken throughout the lough with an average recorded for NO_3_^−^ (0.16 mg/L), NO_2_^−^ (0.12 mg/L) and PO_4_^3−^ (0.22 mg/L). Corresponding concentrations found in the bay at the SGID sources were similar for NO_3_**^−^** (0.017 mg/L, 0.12 mg/L), and NO_2_^−^ (0.05 mg/L, 0.19 mg/L) for low and high tide, respectively. However, the PO_4_^3−^ concentration at the sources was significantly lower (0.05 mg/L and 0.08 mg/L, at low and high tide, respectively). The reason for this apparent decrease in PO_4_^3−^ concentration is not clear and will require further validation and spatial variation studies to identify the extent of these differences. In their previous study, McCormick and Cave et al. [6,8] also found elevated concentrations of nutrients associated with these SGID sources, and, although their values were generally higher than the results obtained in this study, they were of the opinion that the nutrient influx to the bay was primarily coming from SGID freshwater sources associated with Caherglassuan Turlough.

**Figure 15:**
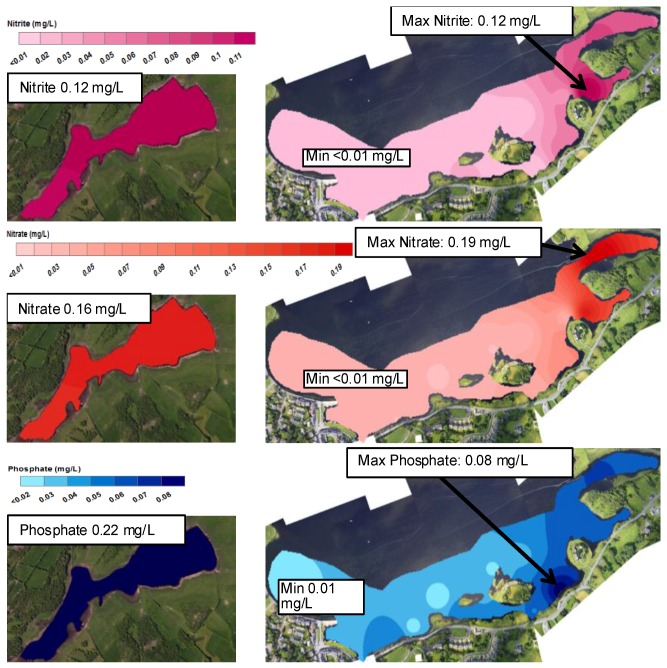
Nitrite (**top**), Nitrate (**middle**) and Phosphate (**bottom**) distribution maps generated from levels found in samples taken at each sampling point within Kinvara Bay and Caherglassuan Turlough at high tide during the sampling campaign (25–28 August 2015).

The colour mapping patterns for nutrient concentration distribution in the inner bay shown in Figure 14 and Figure 15, are strikingly consistent with the salinity and temperature data discussed above, supporting the view that a significant amount of the nutrients in the inner bay comes from the SGID water sources. The stream emerging top right of the images also appears to convey significant amounts of nutrient to the bay (see for example Figure 14). These patterns are not surprising, as the primary source of nutrient in the bay is expected to come from agricultural sources, transported from the turlough along the subterranean conduits and by surface streams.

## 4. Conclusions

This study has generated a large amount of new data, comprising in-situ temperature, salinity and phosphate measurements, together with fly-over IR thermal imaging, coordinated for temporal and spatial coverage. In addition, Landsat 8 satellite-based sea surface temperature measurements were studied for a period of 12 months. The satellite measurements suggested that the water in the inner bay was significantly lower than the rest of the bay, particularly in the area near Dunguaire Castle. In-situ measurements showed that the cooler water was strongly correlated with low salinity, confirming the contention that the cooler regions were due to fresh water emerging from SGID sources in the inner bay. Furthermore, concentration distributions of nutrients (particularly NO_2_^−^ and NO_3_^−^) in the inner bay also exhibited a strongly correlated pattern, consistent with run-off from freshwater sources into the bay. This study suggests that it may be possible to track the dynamics of freshwater-seawater mixing behaviour in the bay using temperature, either by in-situ temperature measurements or by thermal imaging from fly overs or drones, or via satellite remote imaging. Clearly, therefore, the continuing improvements in satellite remote sensing spatial resolution will offer great opportunities to study water mixing dynamics, provided the boundaries for using temperature as a surrogate for salinity can be rigorously established, as this will vary depending on rainfall, tides, wind, sunshine, time of year, the extent to which the freshwater transfer to the sea is subterranean, and other factors. Our results suggest that an ideal strategy is to combine satellite remote SST sensing with an element of in-situ temperature and salinity sensing. The in-situ sensors provide accurate ground-truthing for local calibration of the thermal imaging data, while the satellite imaging offers access to huge spatial coverage. The implication is that the density of in-situ sensing locations could be reduced to a relatively small number of autonomous stations (e.g., instrumented buoys), reducing cost, and leveraging the tremendous potential of satellite data, opening the way to scalable models for implementing wide spatial coverage. Furthermore, the association of nutrient levels with the same locations implies that it may be possible to track the distribution and mixing dynamics of fresh water contamination in the bay at a relatively low cost. While this is an exciting prospect, it must be appreciated that this study was limited in scope, and the experimental data was gathered over a period of several days in a relatively confined region. More extensive studies should be invoked to validate these conclusions, and (if positive) identify the boundaries of the correlations, as these will be dynamic in terms of spatial distribution and the relative impact of various influencing factors.

## Supplementary Materials

The supplementary materials are available online at http://www.mdpi.com/1424-8220/16/9/1402/s1.
