# Supplementary material for: Combining Remote Temperature Sensing with in-Situ Sensing to Track Marine/Freshwater Mixing Dynamics
Source: Sensors (Basel). 2016 Aug 31;16(9):1402. doi: 10.3390/s16091402 (PMC5038680; doi:10.3390/s16091402)
Supplement: Supplementary file 1 [file sensors-16-01402-s001.pdf]

Supplementary Materials: Combining Remote Temperature Sensing with in-Situ Sensing to Track Marine/Freshwater Mixing Dynamics

Margaret McCaul, Jack Barland, John Cleary, Conor Cahalane, Tim McCarthy and Dermot Diamond

In order to derive SST data, raw thermal data values were converted from calibrated digital numbers (DN) to top of atmosphere (TOA) or at-satellite radiance (Equation (1)) [1].

$$L_{\lambda TOA} = M_L\, Q_{Cal} + A_L \tag{1}$$

where: L_λ = TOA spectral radiance (W/(m²·sr·μm)); M_L = Band-specific multiplicative rescaling factor from the metadata; A_L = Band-specific additive rescaling factor from the metadata; Q_{Cal} = Quantized and calibrated standard product pixel values (DN).

TOA radiance (W/(m²·sr·μm)) converted from DN represents the sum of three different fractions of energy including emitted radiation from the Earth's surface as well as upwelling and down welling radiance from the atmosphere. The removal of these atmospheric effects is a critical step because it allows for the representation of emitted radiance solely from the Earth's surface. In lieu of atmospheric data from the date of the satellite overpass, an atmospheric correction tool developed by NASA was applied. Water surface radiances were calculated after site-specific atmospheric parameters transmission; down welling and upwelling radiances were established (Equation (2)) [2].

$$L_{\lambda T} = \frac{L_{\lambda TOA} - L_{\lambda up}}{\tau\varepsilon} - \frac{(1-\varepsilon)}{\varepsilon} L_{\lambda down} \tag{2}$$

where: L_λ = Surface radiance of a blackbody target of kinetic temperature T (W/(m²·sr·μm)); $L_{\lambda TOA}$ = TOA spectral radiance (W/(m²·sr·μm)); τ = Atmospheric transmission (unitless); ε = Emissivity (unitless); $L_{\lambda up}$ = Upwelling or atmospheric path radiance (W/(m²·sr·μm)); $L_{\lambda down}$ = Downwelling or atmospheric path radiance (W/(m²·sr·μm)). Surface water radiance values derived from Landsat TIRS data were then converted in to sea surface temperature (Equation (3)) [2].

$$T_{SS} = \frac{K_2}{\ln(\frac{K_1}{L_{\lambda T}} + 1)} \tag{3}$$

where: T_{SS} = SST (Kelvin); K_1 = Pre-launch thermal calibration constant (W/(m²·sr·μm)); K_2 = Pre-launch thermal calibration constant (Kelvin).

Deriving Temperature Anomaly (TA) Maps

The main objective was to locate potential sites of SGD along the shoreline of Kinvara Bay. These observed surface water temperature patterns aided in the selection of field sites for in-situ verification of aerial and satellite imagery see Figure 3. The accuracy of the absolute temperature values is of less importance in this than the production of inter-comparable thermal anomaly maps than will allow for comparison of SST values from different dates and locations. Sets of Temperature anomaly (TA) maps were generated from each of the SST image collected. TA is defined as the difference between the SST of a pixel and the average SST value for the extent of the Landsat scene (Equation (4)).

$$TA = T_P - T \tag{4}$$

where: TA = Temperature anomaly (°C); T_P = Pixel specific temperature (°C); T = Average temperature value for a specific Landsat scene (°C).

The transformed maps preserve the same spatial pattern of SST but pixel values are rescaled accordingly within the TA maps, from −2.25 to +1.0 °C.

References

1. Landsat8DataUsersHandbook.pdf. Available online: https://landsat.usgs.gov/documents/Landsat8DataUsersHandbook.pdf (accessed on 2 July 2016).
2. Wilson, J.; Rocha, C. Regional scale assessment of Submarine Groundwater Discharge in Ireland combining medium resolution satellite imagery and geochemical tracing techniques. *Remote Sens. Environ.* **2012**, *119*, 21–34.
